# Thrombosis biomarkers: utility in early diagnosis and prognosis of severe pneumonia

**DOI:** 10.1186/s12959-026-00876-3

**Published:** 2026-05-29

**Authors:** Yanru Fan, Rufei Ma, Yuan Zhang, Biao Hu, Huiling Wang, Lan Gao

**Affiliations:** Clinical Lab Department, Henan Provincial People’s Hospital, No. 7, Wei Wu Road, Jinshui District, Zhengzhou China

**Keywords:** Severe pneumonia, Thrombinantithrombin complex, α2-plasmininhibitor- plasmin complex, Soluble thrombomodulin, Tissue plasminogen activator-inhibitor complex

## Abstract

**Background:**

Pneumonia is a major health problem and the most important causes of mortality in all age groups worldwide. We investigated new automation technology to detect plasma biomarkers, including thrombinantithrombin complex (TAT), α2-plasmininhibitor-plasmin complex (PIC), soluble thrombomodulin (sTM), and tissue plasminogen activator-inhibitor complex (t-PAI·C), and evaluated their diagnostic performance and prognostic value for severe pneumonia patients.

**Methods:**

We collected 414 patients date with pneumonia. sTM, t-PAI·C, TAT, PIC were measured by qualitative chemiluminescence immunoassay performed on HISCL analyzers. Other laboratory tests were evaluated on the day of non-severe pneumonia and severe pneumonia diagnosis.

**Results:**

There were significant differences in sTM, t-PAI·C, TAT, PIC (*p* < 0.0001), WBC (*p* = 0.023), PCT (*p* = 0.007) and IL-6 (*p* = 0.002) between the severe pneumonia and non-severe pneumonia groups, Logistic regression analysis showed that sTM (*p* = 0.001), t-PAI·C (*p* = 0.001), TAT (*p* = 0.022), PIC (*p* = 0.000) and APTT (*p* = 0.013) were independent risk factors for severe pneumonia. Logistic regression analysis showed that t-PAI·C (*p* = 0.006) was an independent risk factor for hospital mortality in severe pneumonia. The AUC of sTM combined with t-PAI·C, TAT and PIC on diagnosis of patients with severe pneumonia was 0.868 (95% CI: 0.837, 0.899). Kaplan-Meier survival analysis with a log-rank test showed the in-hospital death rate of severe pneumonia was higher in the high TAT (≥ 5.58ng/mL) level than in group with low TAT (< 5.58ng/mL) level (log rank < 0.029). The same trend with high t-PAI·C was also found in severe pneumonia patients (log rank < 0.021).

**Conclusions:**

The thrombosis markers are helpful for the diagnosis and prognostic assessment of severe pneumonia.

**Supplementary Information:**

The online version contains supplementary material available at 10.1186/s12959-026-00876-3.

## Introduction

Pneumonia is a common acute respiratory infection that affects the alveoli and distal bronchial tree of the lungs. children of < 5 years of age and adults of > 70 years of age are the populations most affected by pneumonia, according to the 2019 GBD (Global Burden of Disease Study) study [1]. At the global level, lower respiratory tract infections, including pneumonia, are the fourth most common cause of death [2]. Systemic coagulation abnormalities, including clotting activation and inhibition of anticoagulant factors, have been observed not only in sepsis but also in pneumonia [3–5].

The pathogenesis of the pneumonia-induced coagulopathy has not yet been fully elucidated, but the mechanisms may overlap in some part to those of bacteria-induced septic coagulopathy/DIC (Disseminated Intravascular Coagulation). The excess production of proinflammatory cytokines, increased levels of damage-associated molecular patterns, the stimulation of cell-death mechanisms and vascular endothelial damage are the major causes of coagulation disorder in any severe infection [6–8]. Since conventional or classical coagulation laboratory tests (CCTs) cannot detect activation of the early rising coagulation system or the inhibition of the fibrinolytic system, coagulation markers that accurately reflect the coagulation status of patients with severe pneumonia are sorely needed. Soluble thrombomodulin (sTM), thrombin-antithrombin complex (TAT), α2-plasmininhibitor-plasmin complex (PIC), and tissue plasminogen activator inhibitor complex (t-PAI·C) have been useful in a variety of diseases [9]; however, there is little research on sTM, TAT, PIC, or t-PAI·C to distinguish between severe and non severe pneumonia.

In this study, we analyzed the coagulation status of patients with pneumonia by determining sTM, TAT, PIC, and t-PAI·C combined with CCTs, and assessed their values in the diagnosis of severe pneumonia and the outcome of it.

## Methods

### Subjects

The study initially enrolled 414 patients with pneumonia in Henan Provincial People’s Hospital between December 2022 and April 2023. This was a single-center prospective study in which patients were diagnosed as having pneumonia. Pneumonia and severe pneumonia were diagnosed in accordance with the Infectious Diseases Society of America/American Thoracic Society Consensus Guidelines on the Management of Community-Acquired Pneumonia in Adults [10]. This study was approved by the Ethics Committee of Henan Provincial People’s Hospital.

### Patients

Demographic and clinical characteristics were collected from the electronic medical record system. Data collection of laboratory results were defined by the results of at admission (within 24 h after admission). The exclusion criteria included (1) patients who had received targeted treatment (e.g., anti-infective agents, anticoagulants/ antiplatelet drugs, or other therapies affecting coagulation function) at other hospitals after symptom onset; (2) patients with congenital coagulation dysfunction; (3) a long-term use of oral or parenteral anticoagulant intake (continuous use of oral anticoagulants for ≥ 3 months before admission); (4) the guardian refusing to sign an informed consent form and a lack of complete data.

Twenty-four healthy individuals (14 male and 10 female) who attended the hospital for a physical examination during the same time period were recruited as controls. The subjects in the control group were included only if they had normal biochemical indices in all routine examinations and no organic diseases. Healthy volunteers were age-matched with enrolled patients, and baseline comparability of age between groups was confirmed by statistical analysis.

### Laboratory test methods

The thrombosis markers sTM, TAT, PIC, and t-PAI·C were determined using matching reagents by a chemiluminescence method on a Sysmex HISCL-5000 chemiluminescence immunoassay analyzer (Sysmex Corporation, Japan). CCTs, mainly include prothrombin time (PT), activated partial thromboplastin time (aPTT), fibrinogen (FIB), and thrombin time (TT), were assessed using a CS-5100 coagulation analyzer from the Sysmex Company in Japan and matching reagents from Siemens Healthineers (Siemens Corporation, German). The timing of coagulation markers measurement was when the patients was admitted to hospital to diagnose pneumonia or severe pneumonia.

### Statistical methods

Data with normal distribution are presented as the mean ± standard deviation, and data without normal distribution are presented as median (M) and inter-quartile range (P25 and P75). Categorical variables were expressed as frequency rates with percentages. Continuous variables were compared using the independent samples T-test (normal distribution), the Mann-Whitney U-test (non-normal distribution) and chi-square test (categorical variables). Logistic regression analysis was used to analyze the independent risk factors of severe pneumonia and mortality outcome due to severe pneumonia following discharge. Kaplan-Meier survival analysis with a log-rank test was performed to estimate the cumulative survival rate of groups with high or low TAT and t-PAI·C level. We implemented the area under the ROC curve (AU-ROC) method to evaluate the sensitivity and specificity of sTM, TAT, PIC, and t-PAI·C in predicting severe pneumonia and the mortality outcome of it following discharge. Statistical analyses were performed by SPSS 17.0 (SPSS, Chicago, IL, USA) and p-value of < 0.05 was considered statistically significant.

## Results

Primary outcomes in the non-severe pneumonia group and severe pneumonia group are shown in Table 1. Patients in severe pneumonia group were significantly higher levels of blood urea (BUN), white blood cell (WBC), C-Reactive Protein (CRP), procalcitonin (PCT), Interle ukin-6 (IL-6), duration in ICU and lower survival rate than the participants in non-severe pneumonia group. Other characteristics like sex, age, Alanine aminotransferase (ALT), total bilirubin (TBIL) and the history of underlying diseases showed no significance between two groups (Table 1).

**Table 1:** Clinical characteristics in this study

Variable	Non-severe pneumonia group(*n* = 220)	Severe pneumonia group(*n* = 194)	*p*
Age (years)	68.9 ± 16.3	62.2 ± 21.6	0.105
Sex, n (%)			0.083
Male	140(63.6)	139(71.6)	
Female	80(36.4)	55(28.4)	
ALT, U/L	20.7(13.8,35.1)	23.5(15.0,35.7)	0.088
TBIL, µmol/L	9.1(6.1,12.4)	10.2(6.9,18.6)	0.074
BUN, mmol/L	5.9(4.3,8.1)	7.5(5.0,12.8)	0.034
WBC, 109 /L	6.56(4.81,8.58)	8.50(6.12,11.56)	0.023
CRP, mg/L	17.64(4.05,66.02)	61.60(25.21,105.39)	< 0.001
PCT, ng/mL	0.06(0.05,0.93)	0.28(0.06,1.34)	0.007
IL−6,pg/mL	7.89(3.32,18.97)	14.98(4.37,42.00)	0.002
Underlying disease (n,%)			
Hypertension	101(45.9)	83(42.8)	0.523
Diabetes mellitus	66(30)	50(25.8)	0.339
Chronic liver disease	9(4.1)	10(5.2)	0.606
Malignancy	25(11.4)	23(11.9)	0.876
Cardiovascular disease	60(27.3)	52(26.8)	0.915
Duration in ICU, days	8.0(2.8,13.5)	17.0(12.0,24.0)	0.005
Outcome at discharge, n (%)			0.000
Survival	214(97.27)	134(69.1)	
Non-survival	6(2.73)	60(30.9)	

Abbreviations: ALT, alanine aminotransferase; TBIL, total bilirubin; WBC, white blood cell count; BUN, blood urea nitrogen; CRP, C-reactive protein; PCT, procalcitonin; IL−6, interleukin-6; ICU, intensive care unit

**Fig. 1:**
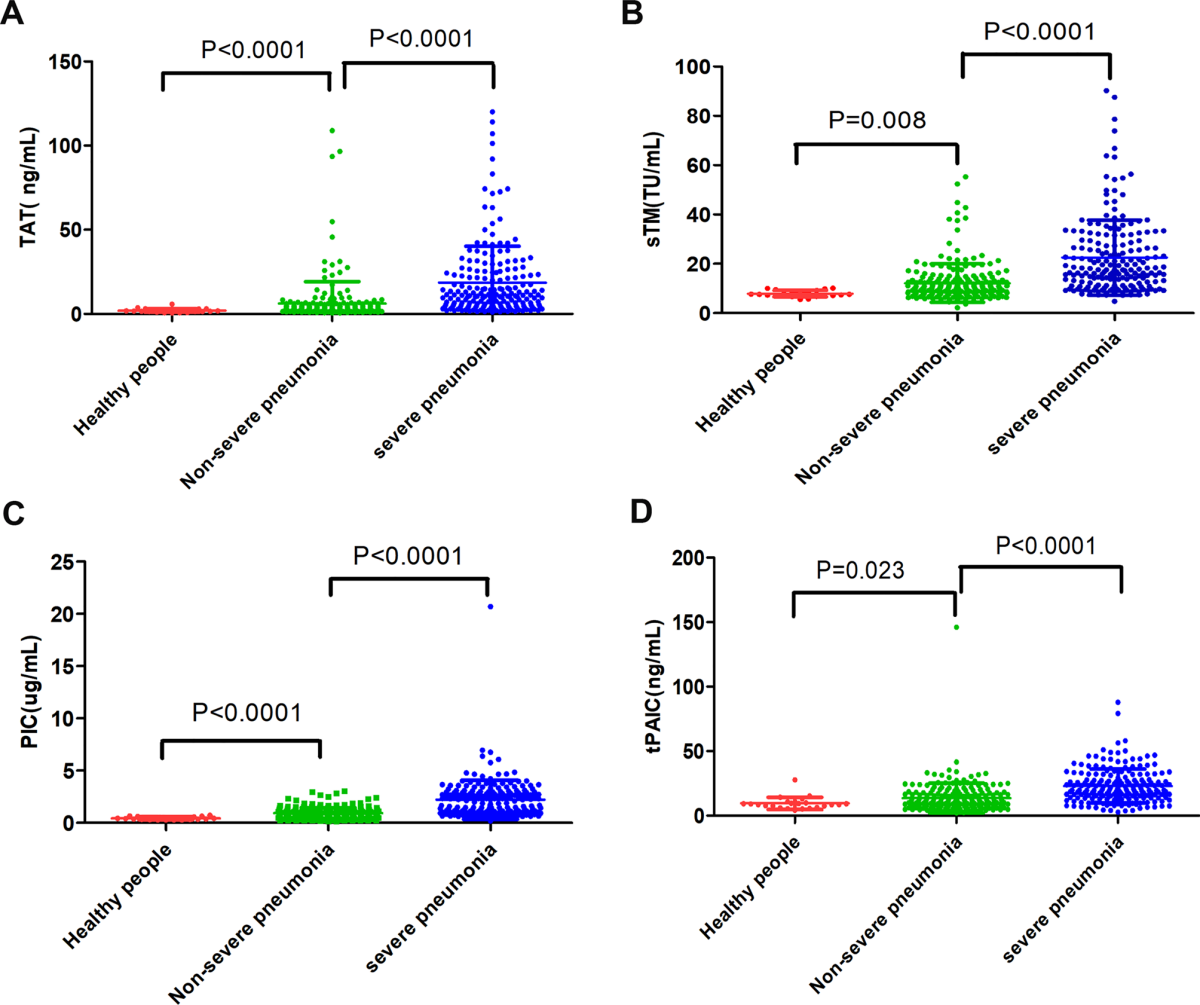
The expression of sTM, TAT, PIC and t-PAI·C in healthy, severe pneumonia and Non-severe pneumonia groups. **A**. Comparison of TAT in three groups. **B**. Comparison of sTM in three groups. **C**. Comparison of PIC in three groups. **D**. Comparison of t-PAI·C in three groups

There were significant differences in sTM, TAT, PIC and t-PAI·C in severe pneumonia and non-severe pneumonia (*p* < 0.001); those differences were also observed between the healthy people and non-severe pneumonia group (*p* < 0.05) (Fig. 1).

The two groups exhibited notable disparities in key coagulation-related parameters, including Plt, PT, INR, aPTT, FDP, and D-dimer, which are closely linked to the activation of the coagulation-fibrinolytic system (*p* < 0.001) (Table 2).

**Table 2:** Secondary outcomes in the Non-severe pneumonia and severe pneumonia groups

Variable	Non-severe pneumonia group (*n* = 220)	Severe pneumonia group (*n* = 194)	*p*
Plt,10^9^ /L	227.76 ± 97.18	193.15 ± 105.54	< 0.0001
PT, s	12.10(11.50,12.90)	13.10(12.30,14.13)	< 0.0001
APTT, s	28.60 ± 4.62	31.42 ± 8.89	< 0.0001
FIB, g/L	4.29 ± 1.55	4.76 ± 1.50	0.700
FDP, ug/ml	2.88(2.50,6.34)	7.38(2.85,18.24)	< 0.0001
D-dimers, mg/ L	0.81(0.42,1.84)	1.75(0.76,5.47)	< 0.0001
INR	1.07(1.02,1.16)	1.17(1.10,1.27)	< 0.0001

Abbreviations: Plt, platelet count; PT, prothrombin time; APTT, activated partial thromboplastin time; FIB, Fibrinogen; FDP, fibrinogen degradation products; INR, international normalized ratio

Logistic regression analysis of sTM, TAT, PIC, t-PAI·C and CCTs in predicting severe pneumonia. Multivariate logistic regression analysis showed that sTM (*p* = 0.001), TAT (*p* = 0.022), PIC (*p* = 0.000), t-PAI·C (*p* = 0.001) and APTT (*p* = 0.013) were independent risk factors for severe pneumonia (Table 3, STable 4).

**Table 3:** Logistic regression analysis of sTM, TAT, PIC, t-PAI·C and CCTs in predicting severe pneumonia

Variable	B	S.E.	Wals	*p*	OR	95% CI.	
						Lower	Upper
sTM, TU/mL	0.052	0.015	11.401	0.001	1.053	1.022	1.085
TAT, ng/mL	0.025	0.011	5.279	0.022	1.025	1.004	1.047
PIC, ug/mL	1.328	0.201	43.846	0.000	3.774	2.547	5.591
t-PAI·C, ng/mL	0.044	0.013	35.561	0.001	1.045	1.018	1.071
APTT, s	0.066	0.026	6.115	0.013	1.068	1.014	1.125

Abbreviations: CCTs, Classical coagulation laboratory tests; TAT, thrombinantithrombin complex; PIC, α2-plasmininhibitor-plasmin complex; sTM, soluble thrombomodulin; t-PAI·C, tissue plasminogen activator-inhibitor complex; APTT, activated partial thromboplastin time

**Fig. 2:**
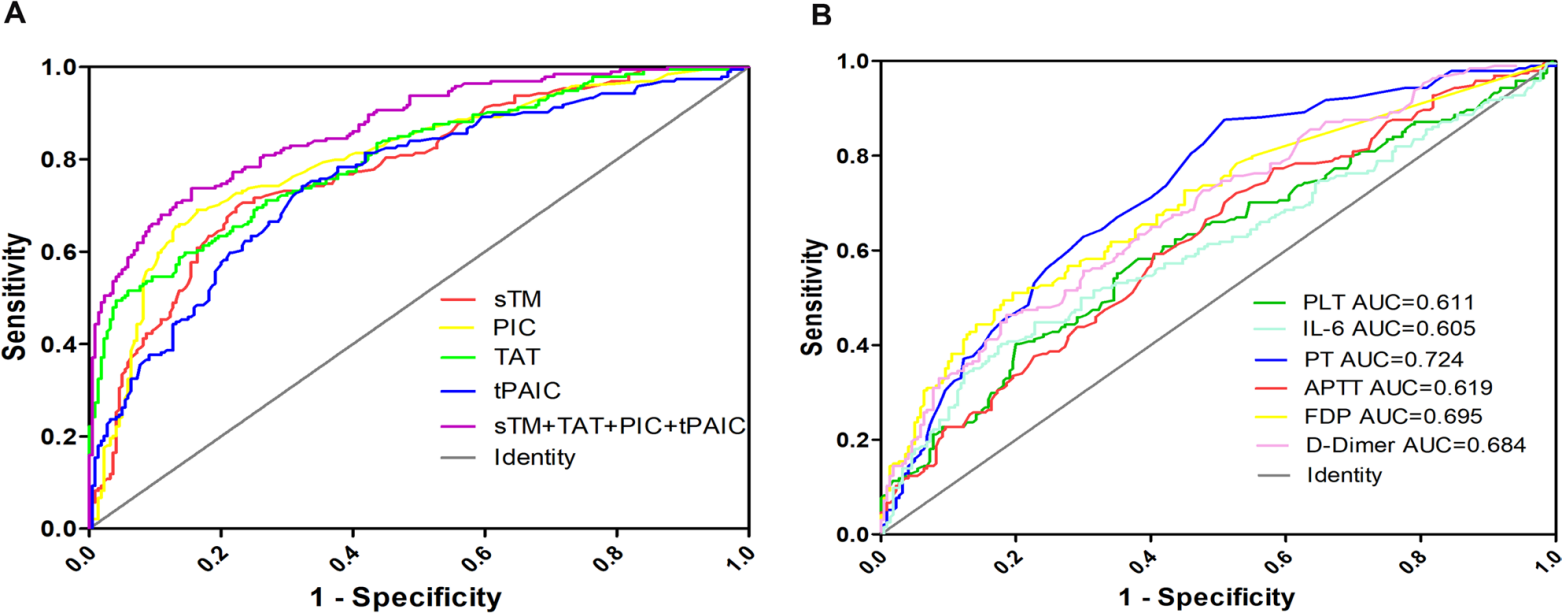
AU-ROC in predicting severe pneumonia. **A**. AU-ROC of independent and combined with sTM, TAT, PIC, t-PAI·C in predicting severe pneumonia. **B**. AU-ROC of other Laboratory testing in predicting severe pneumonia

Receiver operator characteristic (ROC) curve were shown in Fig. 2 to analyze the prognostic value and predicting severe pneumonia.The area under the curve (AUC) of independent and combined with sTM, TAT, PIC, t-PAI·C for severe pneumonia were 0.779 (95% CI 0.734, 0.823, *p* < 0.001), 0.802 (95% CI 0.758, 0.845, *p* < 0.001), 0.804 (95% CI 0.762, 0.845, *p* < 0.001), 0.755 (95% CI 0.708, 0.802, *p* < 0.001) and 0.868 (95% CI 0.837, 0.899, *p* < 0.001) (Table 4; Fig. 2A). AUC of other indicators for severe pneumonia were IL-6 (AUROC = 0.605), PLT (AUROC = 0.611), PT (AUROC = 0.724), APTT (AUROC = 0.619), PDP (AUROC = 0.695), D-Dimer (AUROC = 0.684) (Fig. 2B), CRP (AUROC = 0.704), PCT (AUROC = 0.678) and combined with IL-6, CRP, PCT (AUROC = 0.71) (STable 1).Those results indicated the AUROC values of thrombosis markers combined model for predicting severe pneumonia diagnosis were significantly higher than those of other indicators.

**Table 4:** AU-ROC of thrombosis markers in predicting severe pneumonia

Variable	AUC	SE	95% CI	*P*
sTM, TU/mL	0.779	0.023	(0.734,0.823)	< 0.0001
TAT, ng/mL	0.802	0.022	(0.758,0.845)	< 0.0001
PIC, ug/mL	0.804	0.021	(0.762,0.845)	< 0.0001
t-PAI·C, ng/mL	0.755	0.024	(0.708,0.802)	< 0.0001
TM + TAT+PIC+tPAIC	0.868	0.016	(0.837,0.899)	< 0.0001

Abbreviations: AU-ROC, area under the receiver operating characteristic curve; TAT, thrombinantithrombin complex; PIC, α2-plasmininhibitor-plasmin complex; sTM, soluble thrombomodulin; t-PAI·C, tissue plasminogen activator-inhibitor complex

**Fig. 3:**
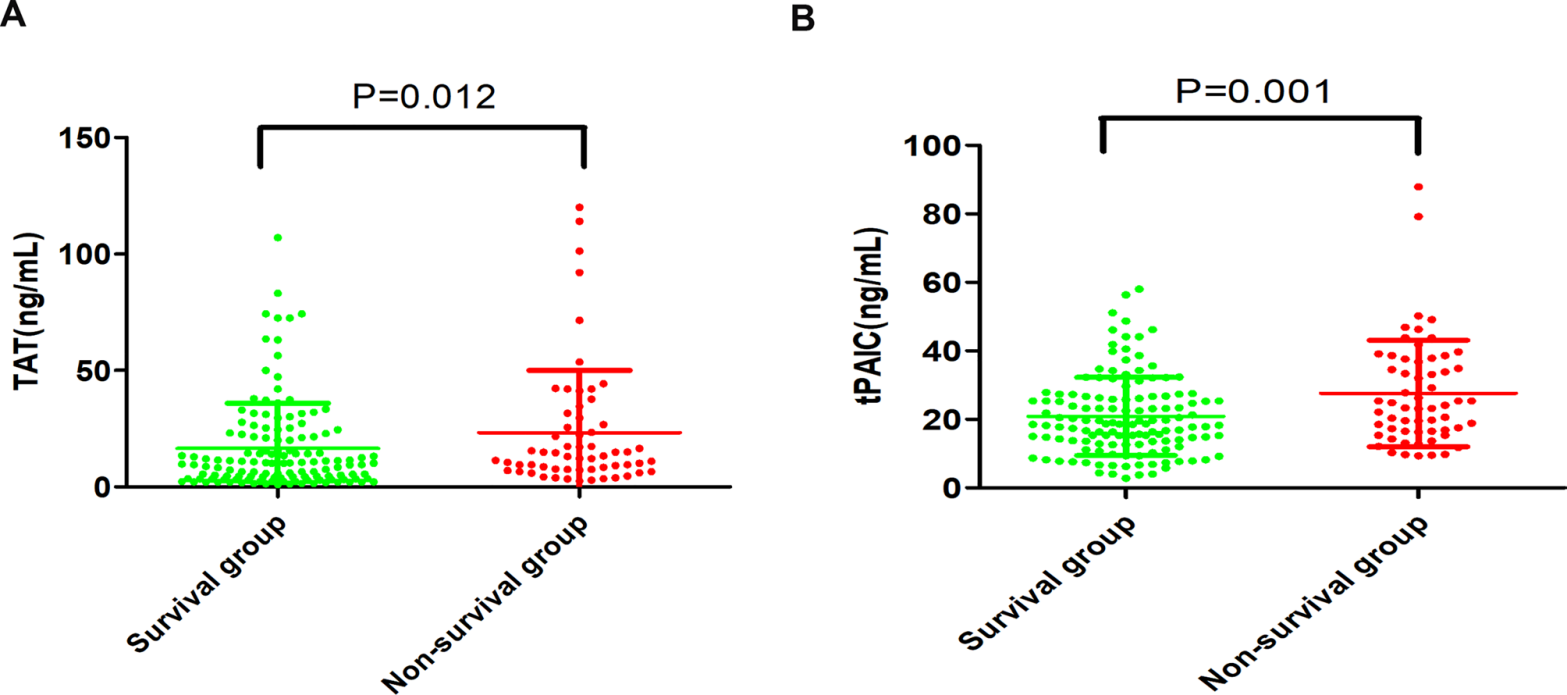
The expression of TAT and t-PAI·C between patients with Survival and Non-Survival group. **A**. Comparison of TAT between patients with Survival and Non-Survival groups; **B**. Comparison of t-PAI·C between patients with Survival and Non-Survival groups

Comparison of outcomes due to death at discharge: 134 severe pneumonia patients survived and 60 died at discharge.There were significant differences in TAT and t-PAI·C between the two groups (*p* < 0.05) (Fig. 3; Table 5).

**Table 5:** Primary and secondary outcomes in the survival and non-survival groups

Variable	Survival group (*n* = 134)	Non-survival group (*n* = 60)	*p*
sTM, TU/mL	17.55(10.78,27.78)	23.25(13.8,32.85)	0.407
TAT, ng/mL	13.20(7.60,26.50)	19.26(3.75,22.68)	0.012
PIC, ug/mL	1.57(0.95,2.80)	2.43(1.09,3.54)	0.078
t-PAI·C, ng/mL	20.86 ± 11.42	27.59 ± 15.52	0.001
Plt,10^9^ /L	203.77 ± 111.81	169.44 ± 86.17	0.036
PT, s	13.1(12.3,14.2)	13.2(12.1,14.1)	0.623
APTT, s	29.9(27.4,33.5)	29.3(27.2,32.5)	0.307
FIB, g/L	4.07(3.18,5.30)	3.98(2.95.5.56)	0.517
FDP, ug/ml	7.7.38(2.70,17.20)	7.85(2.68,19.90)	0.383
D-dimers, mg/ L	1.71(0.81,4.34)	2.19(0.64,5.63)	0.610
INR	1.17(1.10,1.27)	1.19(1.08,1.28)	0.914

Abbreviations: TAT, thrombinantithrombin complex; PIC, α2-plasmininhibitor-plasmin complex; sTM, soluble thrombomodulin; t-PAI·C, tissue plasminogen activator-inhibitor complex; Plt, platelet count; PT, prothrombin time; APTT, activated partial thromboplastin time; FIB, Fibrinogen; FDP, fibrinogen degradation products; INR, international normalized ratio

Logistic regression analysis of TAT, t-PAI·C and CCTs in predicting mortality outcome due to severe pneumonia. Multivariate logistic regression analysis showed that t-PAI·C (*p* = 0.006) was an independent risk factor for mortality outcome due to severe pneumonia (Table 6, STable 5) .

**Table 6:** Logistic regression analysis of TAT, t-PAI·C and CCTs in predicting the mortality outcome due to severe pneumonia at discharge

Variable	B	S.E.	Wals	*p*	OR	95%CI.	
						Lower	Upper
t-PAI·C, ng/mL	0.035	0.013	7.588	0.006	1.035	1.010	1.061

Abbreviations: TAT, thrombinantithrombin complex; t-PAI·C, tissue plasminogen activator- inhibitor complex; CCTs, Classical coagulation laboratory tests

**Fig. 4:**
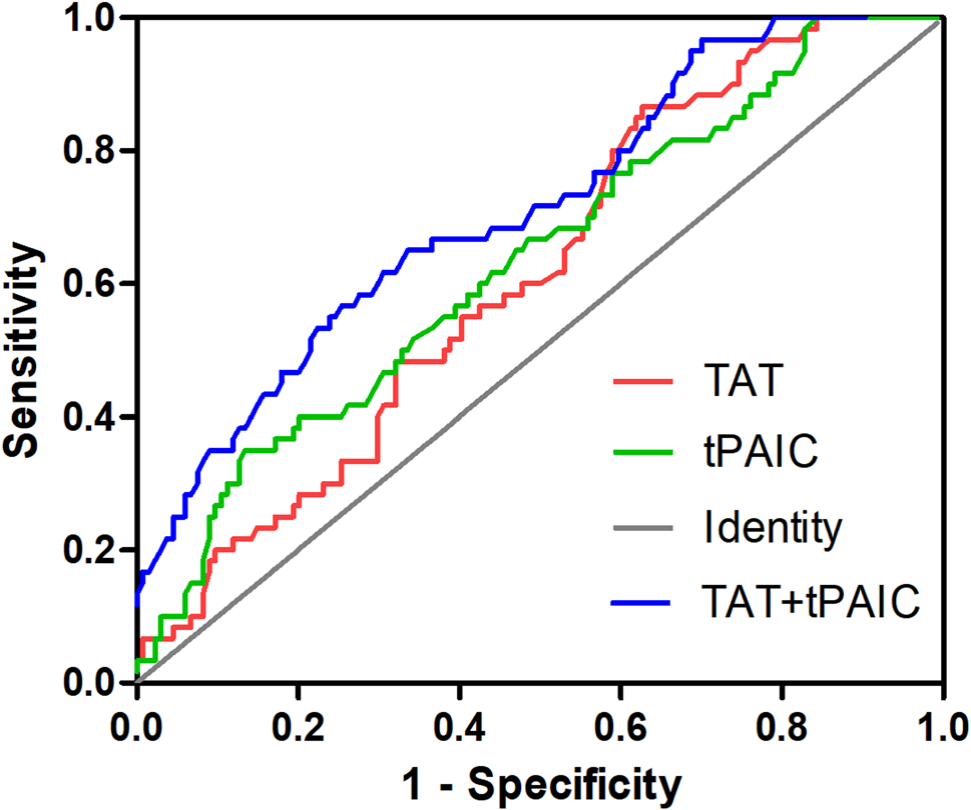
AU-ROC of independent and combined with TAT and t-PAI·C in predicting the mortality outcome due to severe pneumonia at discharge

This study compared the predictive efficacy of the core biomarker panel (TAT + t-PAI-C) with inflammatory markers (IL-6, CRP, PCT) and individual biomarkers via ROC curve analysis. AUC of independent and combined with TAT and t-PAI·C for death of severe pneumonia were 0.614 (95% CI 0.532, 0.695, *P* = 0.012), 0.643 (95% CI 0.550, 0.718, *P* = 0.003) and 0.656 (95% CI 0.573, 0.738, *P* = 0.001) (Table 7; Fig. 4). The results showed that the AUROC values of this combined model for predicting death of severe pneumonia were significantly higher than those of IL-6 (AUROC = 0.622), PCT (AUROC = 0.598) with statistically significant differences (*P* < 0.05). However, ROC analysis showed that the AUC-ROC values of CRP for predicting severe pneumonia in-hospital mortality were 0.538, with no statistically significant difference (*P* > 0.05) (STable 2).

**Table 7:** AU-ROC of independent and combined with TAT, t-PAI·C in predicting the mortality outcome due to severe pneumonia at discharge

Variable	AUC	SE	95% CI	*p*
TAT, ng/mL	0.614	0.041	(0.532,0.695)	0.012
t-PAI·C, ng/mL	0.634	0.043	(0.550,0.718)	0.003
TAT+tPAIC	0.656	0.042	(0.573,0.738)	0.001

Abbreviations: TAT, thrombinantithrombin complex; t-PAI·C, tissue plasminogen activator- inhibitor complex

**Fig. 5:**
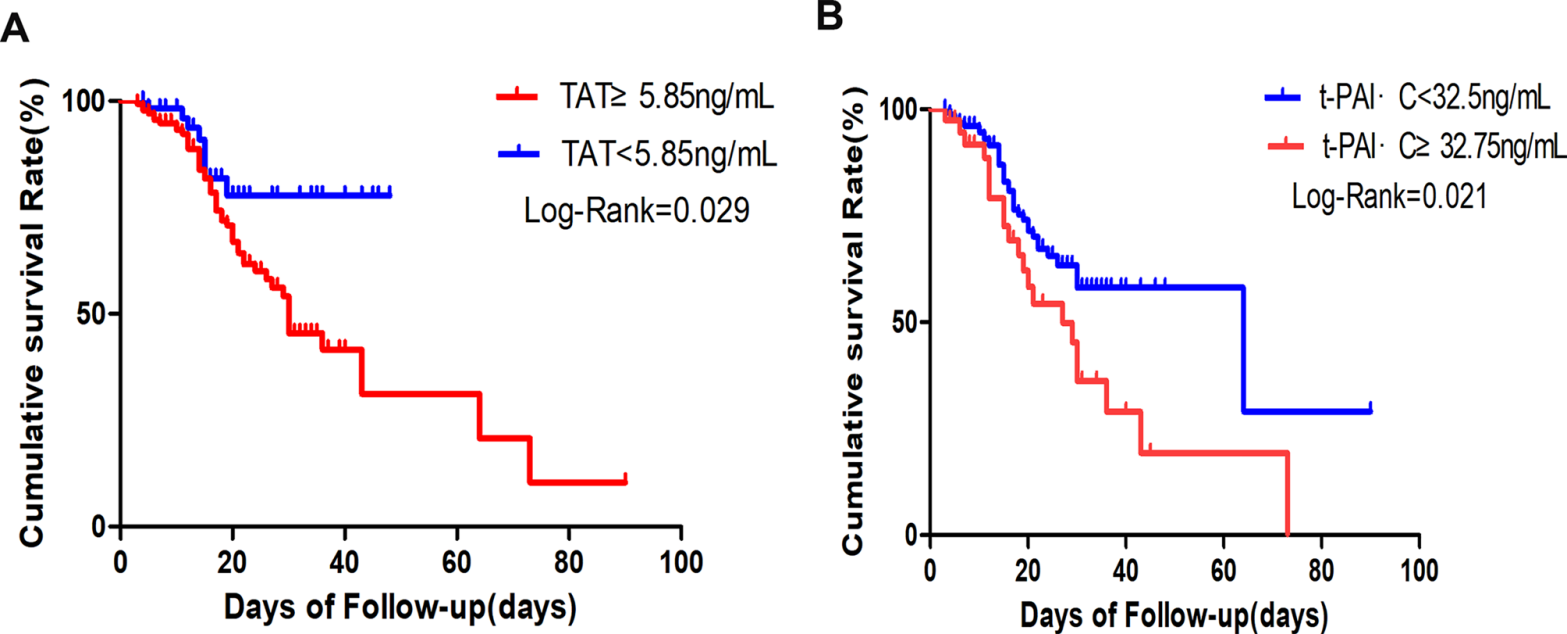
Kaplan-Meier plots showing the cumulative survival rate of severe pneumonia patients who were stratified into two groups according to plasma TAT and t-PAI·C cutoff point at baseline. **A**. The cumulative survival rate of severe pneumonia patients by plasma TAT. **B**. The cumulative survival rate of severe pneumonia patients by plasma t-PAI·C

Baseline characteristics of severe pneumonia patients were divided into two groups by low and high TAT levels (TAT < 5.85 ng/mL and TAT ≥ 5.85 ng/mL) according to the cut-off value determined in the ROC curve (Fig. 4).Cumulative survival rate curves between two groups categorized by TAT cut-off value were shown in Fig. 5A. During follow-up, the in-hospital death rate was higher in the high TAT level than in group with low TAT level (log rank < 0.029). The same trend with high t-PAI·C was also found in patients with severe pneumonia(log rank < 0.021) (Fig. 5B).

## Discussion

Pneumonia remains one of the most important causes of mortality. It is the first cause of respiratory death all over the world, excluding lung cancer [11]. Studying hospital admissions for moderate to severe pneumonia has been one way to understand the characteristics and the impact of this disease [12, 13]. Although it has been reported that the diagnosis of pneumonia relies on symptoms, clinical features and radiology, those cannot effectively distinguish severe pneumonia. Thus, this study is intended to seek potential molecular markers for diagnosing and evaluating prognosis of severe pneumonia.

In this study, we observed significantly elevated sTM, TAT, PIC and t-PAI·C expressions in severe pneumonia groups compared with healthy and non-severe pneumonia. sTM, a transmembrane glycoprotein, is abundant on all endothelial surfaces [14]. TAT directly reflects the activation of the coagulation system and can be used as a marker of coagulation initiation [15]. The half-life of activated plasmin is very short; and plasmin reacts rapidly with fibrinolytic system α2 plasmin inhibitor to combine 1:1 and thereby form PIC, which then reflects the early rise in plasmin and measurement of which can be used to evaluate the degree of fibrinolytic activation in vivo [16]. In addition, the fibrinolytic system is principally mediated by plasminogen activator inhibitor-1 (PAI-1) and PIC, and thrombin activator fibrinolysis inhibitor (TAFI) leads to fibrinolytic inhibition. PAI-1 forms t-PAI·C by combining with tissue plasminogen activator (t-PA) to inactivate it, thereby inhibiting the dissolution of fibrin [17]. The emergence of t-PAI·C can provide an early indication of the occurrence of fibrinolytic inhibition, and is closely related to endothelial cell injury [18]. Interestingly, this study revealed that sTM, TAT, PIC, t-PAI·C and APTT were independent risk factors for severe pneumonia. Pneumonia is an infection of the lung causing exudative fluid to accumulate in the pulmonary parenchyma, compromising respiratory function. Pneumonia is by far the most common cause of sepsis [19, 20], which has been defined by an international task force as organ dysfunction due to a dysregulated host response to infection that is severe enough to be life threatening [21]. Pneumonia is distinguished from other forms of sepsis by the location; when the infection causing dysregulated host response is in the lungs, then it is pneumonia. Sepsis leads to endothelial cell damage, activation of the procoagulant system, and inhibition of anticoagulant substances such as antithrombin (AT), protein C (PC), and TFPI. In addition, alveolar macrophages, lung epithelial cells, neutrophils and so on can undergo dramatic transcriptional remodeling, such as NF-κB and STAT3 signal pathway, in response to infection or infectious stimuli, and such activity is elicited by both pathogen- and host-derived mediators, as enabled by a wide gamut of receptors for pathogen-associated molecular patterns (PAMPs), damage-associated molecular patterns (DAMPs), cytokines, and other immunomodulatory agents [22–25]. Thus, IL-6, CRP and CCTs highly expressed in severe pneumonia. However, AUC of independent and combined with sTM, TAT, PIC, t-PAI·C is better than IL-6 and other indicators for severe pneumonia. Although inflammatory markers such as IL-6, CRP, and PCT can indicate the severity of infection, they cannot specifically reflect the activation of the coagulation system, a key link closely associated with severe pneumonia complications (e.g., thrombosis, multiple organ failure) and mortality. In this study, the thrombosis biomarker panel can serve as a complementary tool for improving the accuracy of clinical decision-making in severe pneumonia.

Besides the effect of four thrombosis markers on diagnosing severe pneumonia, we also elucidated that those markers predict mortality from severe pneumonia. It is well documented that sTM, TAT, PIC, and t-PAI·C can be utilized to evaluate the changes in coagulation status from the critical environments of many pathophysiological mechanisms that underly Sepsis-induced coagulopathy (SIC) — including endothelial cell injury, early-coagulation activation, and fibrinolytic-system inhibition. TAT, PIC, and t-PAI·C be detected in sepsis-related DIC to evaluate the status of coagulation disorders [26]. Moreover, sTM, TAT, PIC and t-PAI·C have been reported to be the clinical value of in diagnosis and outcome of pediatrics sepsis and pSIC [27]. In this study, we observed remarkably elevated expression of TAT and t-PAI·C in non-survival group, whereas logistic regression analysis revealed t-PAI·C was an independent risk factor for mortality outcome due to severe pneumonia. Interestingly, the higher TAT and t-PAI·C level, the higher in-hospital death rate. The plasma level of t-PAI·C is known to be related with organ failure, and sTM can reflect poor clinical outcomes [28]. PIC, however, is not a significant prognostic factor [29]. As for TAT, some reports showed that TAT levels were significantly higher in patients with poor outcomes than those without [30]. This study confirmed the value of sTM, TAT, PIC, t-PAI·C in the early diagnosis of severe pneumonia and prediction of in-hospital mortality, providing a reliable reference for clinically identifying high-risk patients by systematically analyzing the expression characteristics of coagulation-fibrinolytic biomarkers (sTM, TAT, PIC, t-PAI·C) at hospital admission and optimizing intervention strategies. Future studies could expand the sample size for multi-center prospective validation to further clarify the application efficacy of this biomarker panel in different clinical scenarios.

This study has several limitations that need to be addressed in future research.This was a single-center retrospective/prospective study with a limited sample size and single source of participants, which may introduce selection bias. The generalizability of the results requires further verification by multi-center, large-sample prospective studies. Secondly, Coagulation-fibrinolytic biomarkers and classical coagulation indicators were only measured once at hospital admission. The dynamic changes of biomarkers during disease progression were not captured, and the association between biomarker fluctuations, disease evolution, and treatment response cannot be clarified. Finally, this study only verified the diagnostic and prognostic value of the biomarker panel, but did not explore the specific mechanism of these biomarkers in the “inflammation-coagulation crosstalk” of severe pneumonia. Subsequent studies should combine basic experiments to clarify the molecular regulatory pathways.

## Conclusions

In summary, we have elucidated the role of thrombosis markers in diagnosing severe pneumonia patients. Importantly, both high expression of TAT and t-PAI·C were associated with in-hospital death in patients with severe pneumonia. These findings indicate that thrombosis markers might be potential molecular markers for diagnosing and evaluating prognosis of severe pneumonia. 

## Supplementary Information

Below is the link to the electronic supplementary material.

Supplementary Material 1

## Data Availability

No datasets were generated or analysed during the current study.

## Abbreviations

TAT: Thrombinantithrombin complex
PIC: α2-plasmininhibitor- plasmin complex
sTM: Soluble thrombomodulin
tPAIC: Tissue plasminogen activator-inhibitor complex
CCTs: Classical coagulation laboratory tests
WBC: White blood cell
CRP: C-reactive protein
PCT: Procalcitonin
IL-6: Interleukin-6
APTT: Activated partial thromboplastin time
PT: Prothrombin time
AUC: Area under curve
ROC: Receiver operating characteristic curve
TAFI: Thrombin activator fibrinolysis inhibitor
