# Supplementary material for: Thrombosis biomarkers: utility in early diagnosis and prognosis of severe pneumonia
Source: Thromb J. 2026 May 29;24:70. doi: 10.1186/s12959-026-00876-3 (PMC13435470; doi:10.1186/s12959-026-00876-3)
Supplement: Supplementary file 1 [file 12959_2026_876_MOESM1_ESM.docx]

STable 1. AU-ROC of **Inflammatory infection markers** in predicting severe pneumonia.

| Variable | AUC | SE | 95% CI | P |
| --- | --- | --- | --- | --- |
| CRP, mg/L | 0.704 | 0.025 | (0.654,0.754) | <0.0001 |
| PCT, ng/mL | 0.678 | 0.027 | (0.626,0.730) | <0.0001 |
| IL-6,pg/mL | 0.605 | 0.028 | (0.549,0.660) | <0.001 |
| CRP+PCT+IL-6 | 0.710 | 0.025 | (0.661,0.760) | <0.0001 |

Abbreviations: CRP, C-reactive protein; PCT, procalcitonin; IL-6, interleukin-6

STable 2. AU-ROC of **Inflammatory infection markers** in the mortality outcome due to severe pneumonia at discharge.

| Variable | AUC | SE | 95% CI | P |
| --- | --- | --- | --- | --- |
| CRP, mg/L | 0.538 | 0.044 | (0.451,0.625) | 0.397 |
| PCT, ng/mL | 0.598 | 0.046 | (0.508,0.687) | 0.030 |
| IL-6,pg/mL | 0.622 | 0.043 | (0.587,0.757) | <0.001 |

Abbreviations: CRP, C-reactive protein; PCT, procalcitonin; IL-6, interleukin-6

STable 3. Clinical characteristics in this study.

| Variable | Heathly group  (n=24) | pneumonia group(n=414) | p |
| --- | --- | --- | --- |
| Age (years) | 56.4±23.7 | 65.1±18.8 | 0.09 |

STable 4 Logistic regression analysis of CCTs in predicting severe pneumonia.

| Variable | B | S.E. | Wals | p | OR | 95%C.I. | |
| --- | --- | --- | --- | --- | --- | --- | --- |
|  |  |  |  |  |  | Lower | Upper |
| PT, s | 0.096 | 0.052 | 3.448 | 0.063 | 1.101 | 0.995 | 1.218 |
| TT, s | 0.290 | 0.169 | 2.958 | 0.085 | 0.748 | 0.537 | 1.041 |
| FIB, g/L | 0.000 | 0.001 | 0.201 | 0.654 | 0.999 | 0.996 | 1.002 |

Abbreviations: CCTs, Classical coagulation laboratory tests; PT, prothrombin time; TT, prothrombin time; FIB, Fibrinogen

STable 5. Logistic regression analysis of TAT, t-PAI·C and CCTs in predicting the mortality outcome due to severe pneumonia at discharge.

| Variable | B | S.E. | Wals | p | OR | 95%C.I. | |
| --- | --- | --- | --- | --- | --- | --- | --- |
|  |  |  |  |  |  | Lower | Upper |
| PT, s | 0.100 | 0.053 | 3.560 | 0.069 | 1.105 | 0.996 | 1.225 |
| TT, s | 0.034 | 0.012 | 2.517 | 0.114 | 1.012 | 0.997 | 1.025 |
| FIB, g/L | 0.350 | 0.185 | 3.002 | 0.103 | 0.828 | 0.629 | 1.057 |
| APTT, s | 0.049 | 0.186 | 3.100 | 0.083 | 0.894 | 0.847 | 1.069 |
| TAT, ng/mL | 0.009 | 0.007 | 1.740 | 0.187 | 1.009 | 0.996 | 1.023 |

Abbreviations: CCTs, Classical coagulation laboratory tests; PT, prothrombin time; TT, prothrombin time; FIB, Fibrinogen; APTT, activated partial thromboplastin time;TAT, thrombinantithrombin complex
